# The Decrease in Traumatic Brain Injury Epidemics Deriving from Road Traffic Collision Following Strengthened Legislative Measures in France

**DOI:** 10.1371/journal.pone.0167082

**Published:** 2016-11-28

**Authors:** Thomas Lieutaud, Blandine Gadegbeku, Amina Ndiaye, Mireille Chiron, Vivian Viallon

**Affiliations:** 1 UMRESTTE, IFSTTAR, UMR T_9405, Bron, Université de Lyon, Lyon, France; 2 Inserm U10128, CNRS UMR5292, Lyon Neuroscience Research Center, team TIGER, Université Claude Bernard Lyon 1, Université de Lyon, Lyon, France; Beihang University, CHINA

## Abstract

**Background:**

Since 2002, France has been strengthening legislation on road traffic. This study is intended to evaluate the changes in Traumatic Brain Injury (TBI) incidence and mortality resulting from Road Traffic Collision (RTC) in the two 6-year periods before and after 2002.

**Methods:**

We used a Registry of all RTC casualties in the Rhône Department of France. Each casualty was coded according to the Abbreviated Injury Scale (AIS). The study describes changes in demographic variables, TBI (AIS ≥ 2) incidence and mortality, other body lesions (AIS ≥ 3) associated with TBI, road user types, seatbelt and helmet wearing.

**Findings:**

RTC casualty occurrences decreased by 21% (from 64,312 to 50,746) during the period after 2002. TBI occurrence accounted for 8.6% and 6.7% of all RTC in both periods. This corresponds to a reduction of TBI casualty incidence (-42%), which was much more pronounced than RTC casualty incidence (-25%) (p < 0.0001). Severe and critical TBI (AIS-4 and -5) incidences were reduced by half as much (-21%), compared to TBI incidence. TBI mortality rate (among population) and lethality (among TBI related to RTC casualties) decreased 56% and 23%, respectively. This reduction particularly affected car occupants and victims who deceased. TBI incidence decreased 43% in all 10-year age classes until 60 on average, this decrease declining with age in the period after 2002. After adjustment for age, sex, road user types, and severity of lesions at the head and other body regions, logistic regression analysis displayed a protective effect of the period following 2002, on the risk of death after RTC-related TBI.

**Interpretation:**

The greater reductions in the incidence, severity and mortality of TBI when compared with the reduction of casualty incidence have mainly affected car users. These results should be attributable to the improvements in standards of care, primary safety of the car fleet and general road architecture safety. However, the increased reduction in the TBI epidemics in France, when compared to those observed in other developed countries for the same periods, suggests that the effects should be strongly attributable to changes in road user behaviour induced by law enforcement. The at-risk groups for TBI after RTC are now two-wheel users (motorized or not) and individuals over 60 years of age.

## Introduction

Traumatic Brain injury (TBI) is one of the leading causes of injury, death and disability in western countries. For instance, it affects 1.7 million US civilians, every year, including 52,000 fatalities, i.e. one-third of all injury-related deaths. The incidence of TBI-related death is 18.4/100,000 population per year (1997–2007), with one-third due to Road Traffic collisions (RTC) [1]. Road crashes now represent the third-highest cause of acquired disease worldwide with a 45% increase in the share of death and a 34% increase in the proportion of disability-adjusted life year [2] in the last decade. In developed countries, however, this share is lower, with trends of around 10% decrease, in the USA for example [3]. Changes in the incidence of trauma, TBI and mortality over time may have multiple causes. Some authors have identified risk factors for trauma-related death and TBI, such as the consumption of cannabis [4] or other psychoactive and non-psychoactive [5] medications, while other factors—speed control [6], reduced drink-driver thresholds [7], and improved hospital emergency care [8]—have been linked to the decrease in said trauma and deaths [2–4]. As RTCs generated half of the severe TBIs in France in the 1990s [9], with figures higher than in many other European countries [10,11], an epidemiological database of all RTC casualties has been available since 1995 in the Rhône area of France [12]. Using this database, we have observed that, subsequent to legal decisions made since 2002, road trauma incidence decreased 25% in the period 2003–2008 compared to the previous 6-year period (1996–2001), whereas Spinal Cord Injury (SCI) incidence was not on the decrease [13].

In this study, we hypothesize that RTC-related trauma and TBI incidences may differ before and after law enforcement, which alters user behaviours, the associated injuries and the mortality rates.

## Methods

This study uses recorded data from the Rhone Registry [12]. In short, the Registry covers the Rhône Department (≈ 1.6 million inhabitants, 528 hab/km^2^) and has been approved by the health authorities (National Registry Committee and National Commission for Information Technology and Civil Liberties N° 999211). The Registry collects the demographic characteristics of each road crash casualty, the type of road user, and a description of the body injuries sustained (S1 Fig). The inclusion criteria are as follows: an RTC involving at least one vehicle (motorized or not) occurring in the Rhône area, requiring institutional health care activity from one of the 245 healthcare structures cooperating together, including pre-hospital primary care teams and forensic medicine institutes. Each injury was coded by one of the authors (AN), using the Abbreviated Injury Scale (AIS version 90) [14]. Selections of TBI victims were based on the presence of any lesion at the head with an AIS score ≥ 2 (corresponding to at least a loss of consciousness of any duration). Severe, critical and maximal TBIs were defined, respectively, as AIS-4, AIS-5 and AIS-6 in the head region. Injury severity score (ISS) was calculated using the square of the AIS score for the most severe lesion observed in three other body regions. To best describe lesions of the other 6 body regions concurring with a TBI, the lesion with the highest AIS score ≥ 3 (Maximum AIS or MAIS) for each body region was retained.

Road users were divided into five categories: car occupants, powered two-wheeler (PTW) users, cyclists, pedestrians, and other road users (rollers, skate boarders, bus occupants or drivers, truck drivers etc.). The use of seatbelts or helmets was also analysed for car occupants and PTW users, respectively. Death was medically certified at the scene or noted in medical charts during hospital stay.

### Observation periods

The study aimed to investigate the trends in the incidence, mortality and lethality rates of TBI following an RTC in the 1996–2008 period. We divided the period into two distinct 6-year sub-periods (1996–2001 and 2003–2008), based on the national survey, which showed a significant change before and after the year 2002 (this year was excluded) (justifications in 15). The database was set up in 1995, and we have exploitable data from 1996 onwards. This meant that we had a 6-year period until the implementation of the new road legislation in 2002. We reproduced this 6-year time-frame to study the period after the legislative change, thus limiting it to the period 2003–2008.

### Statistical analysis

Comparisons were made using SAS software V9.1. Continuous data were analysed using the T-test, the categorical data using the Chi-square test or the Fisher test, as required. A logistic regression was conducted to assess the odds ratio of the period 2003–2008 on the risk of death following RTC-related TBI, after adjustments for age, sex, road user type and head and other body region lesion severity. In all analyses, p < 0.05 was considered as statistically significant.

## Results

### A more pronounced reduction in TBI occurrence/incidence than in RTC

The number of casualties decreased 21% (64,312 vs 50,746) in the period 2003–2008 in comparison to the period 1996–2001, this decrease being more marked for the number of RTC-related TBI (-39%, 5,558 vs 3,375) (Table 1).

**Table 1:** Trends of figures, incidence and mortality rates of road crash trauma and RTC-related TBIs regarding the TBI-severity in the two observation periods, before and after law enforcement in France.

Period		1996–2001			2003–2008			Trends between periods		p
Population (Inhabitants)		9 451 938			9 959 966					
		N	Incidence/1,000,000		N	Incidence/1,000,000				
All casualties		64 312	6 804.1		50 746	5 095.0		-25%		< 0.001
Casualties with a TBI		5 558	588.0		3 375	338.9		-42%		< 0.001
All Deaths		792	83.8		508	51.00		-39%		<0.001
Deaths with a TBI		514	54.4		241	24.2		-56%		< 0.001
***Severity of brain lesions***										
Mild to moderate TBI	MAIS 2	4 303	455.3	506.7	2 527	253.7	281.7	-44%	-44%	< 0.001
	MAIS 3	486	51.4		279	28.0		-46%		< 0.001
Severe TBI	MAIS 4	421	44.5	68.7	375	37.7	54.5	-15%*	-21%*	0.0178
	MAIS 5	228	24.1		168	16.9		-30%		0.0004
Fatal TBI	MAIS 6	120	12.7		26	2.6		-80%*		< 0.001

* means p < 0.05 when compared to TBI trends between periods

The share of TBI among all casualties decreased from 8.6% in the period 1996–2001 to 6.7% in the period 2003–2008 (-23%; p <0.001). Correcting the results for population growth in the Rhone Department allowed us to calculate the reductions of RTC casualty incidence (-25%) and RTC-related TBI incidence (-42%), which were both highly significantly reduced in the later period (p < 0.001). However, the incidence reduction of RTC-related TBI was not homogenous with a smaller but still significant incidence reduction among severe AIS-4 TBI (-15%, p = 0.018) or critical AIS-5 (-30%, p = 0.004), compared to those observed for minor (AIS-2) and serious (AIS-3) TBI (-44%, p < 0.001), but also for maximal (AIS-6) TBI (-80%, p < 0.001) (Table 1). Mortality rate after RTC was 8.4/100,000 in the period 1996–2001 dropping to 5.1/100,000 population in the recent period (-39%, p < 0.001).

### A more pronounced reduction of lethality for moderate TBI

As the number of deaths after RTC decreased 36% (792 to 508, Table 1), the share of TBI among the deceased dropped from 65% (514 among 792) to 47% (241 among 508) in the period 2003–2008. Parallel to these results, the number of deaths in the case of a TBI related to an RTC was reduced 53% (514 to 241), and the corresponding incidence decreased even more (56%; p < 0.0001) in the period 2003–2008 (Table 1). The TBI lethality rate (deaths among TBI casualties) dropped 22.8%, from 9.2% to 7.1% in the period 2003–2008 (p < 0.0001) (Table 2).

**Table 2:** Trend of the figures, incidence, mortality and lethality rates among RTC-related TBI victims between the two observation periods

Periods	1996–2001			2003–2008			Trends			p
	TBI Survivors	TBI Deceased	Letality Rate	TBI Survivors	TBI Deceased	Letality Rate	TBI Survivors	TBI Deceased	Letality Rate	(letality rate)
TBI Casualties	5 044	514	9.2%	3 134	241	7.1%	-38%	-53%	-23.7%	0.0006
Incidence (/1 000 000)	533.6	54.4		314.7	24.2		-41%	-56%		< 0.001
***TBI Severity***										
MAIS2	4 266	36	0.84%	2 512	15	0.59%	-41%	-58%	-29.0%	0.3267
MAIS3	375	111	22.8%	245	34	12.2%	-35%	-69%	-46.6%	0.0004
MAIS4	303	118	28.0%	293	82	21.9%	-3%	-30%	-22.0%	0.0550
MAIS5	99	129	56.8%	84	84	50.0%	-15%	-34%	-11.6%	0.2318
MAIS6	-	120	100%	-	26	100%	-	-78%	-	-

The decrease in incidence and lethality rates varied among head AIS severity groups. Although there was a deep decrease in the incidence of AIS 2 TBI related to casualties, the mortality rate in this group did not change significantly. This can be explained by their low mortality risk, death-related trauma generally deriving from injuries in other body regions. A significant reduction in the mortality rate was observed for casualties with AIS-3 score in the period 2003–2008 (Table 2).

Only a trend towards significance was displayed for AIS-4, and to a lesser extent for AIS-5 TBI mortality rates (p = 0.055 and 0.2318 respectively).

### A more pronounced reduction of TBI casualties among non-survivors and car occupants

**Fig 1:**
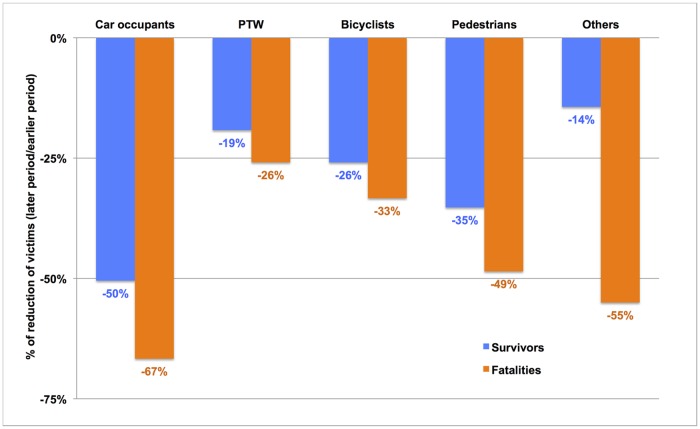
Trends of the TBI victim numbers according to their road user type. Trends of road user types figures (Number in the period 2003-2008/number in the period 1996–2001) according to their deceased (orange) or survivor (blue) status

In the period 2003–2008, all road user types exhibited a reduction in TBI figures related to a road crash, particularly among those non-surviving to the crash (Fig 1). This reduction was most marked for car occupants, who displayed a one-third reduction of RTCs and a 52% reduction of RTC-related TBIs (Table 3).

**Table 3:** Trends of RTC figures and RTC-related TBI regarding their severity among the different road user types in the observation periods (Victims without injuries encoded in the Registry were removed from the analysis (83 and 50 RTC victims without TBI in both periods respectively; all of these victims died at the scene)

Periods	1996–2001				2003–2008				Trend of TBI	p (for TBI trend)
*TBI severity*	None*	*Severe*	*Fatal*	All TBI	None*	*Severe*	*Fatal*	All TBI		
Car Occupants	30672	283	61	**2747**	20830	177	10	**1312**	-52%	< 0.001
Power Two-Wheelers	10856	116	18	**971**	11745	138	7	**779**	-20%	< 0.001
Pedestrians	4982	168	26	**922**	4172	138	7	**579**	-37%	< 0.001
Bicyclists	7813	63	6	**688**	6780	65	2	**508**	-26%	0.0086
Others	4348	19	9	**230**	3794	25	0	**197**	-14%	0.8912

*Victims without injuries encoded were removed from the analysis i.e. 83 and 50 RTC victims without TBI in both periods respectively died at the scene.

At the opposite end, the number of PTW casualties was the only one to have risen in the period 2003–2008. However, although the reduction in TBI injuries was also observed for PTW and pedal cyclists, there was a significant increase in the figure of PTW users and cyclists sustaining severe TBI in the period 2003–2008 when data are pooled together (Fig 1, Table 3).

### Rise of associated injuries among fatalities

The main body region with MAIS ≥ 3 lesions associated with a RTC-related TBI is the thorax. This type of lesion affects 7.4% of the survivors and 54% of the deceased. Abdominal and spinal injury MAIS ≥ 3 occurred in 1.5 and 1.6% among TBI victims who survived the RCT, and in 17.6% and 10.2% among those who died, respectively. The percentage of TBI victims with MAIS ≥ 3 injuries in other body regions than the head was 6 to 7 times higher among victims who died than among the survivors (74.3% vs 16.0% in the period 1996–2001, against 81.3% vs 15.5% in the period 2003–2008). This figure increased significantly among the deceased in the period 2003–2008 (+9.4%, p = 0.0341) (Table 4), with a significant increase for abdominal and spinal injuries in the period 2003–2008 (+60.0%; p = 0.0138 and +177.9%; p < 0.0001 respectively). In the same period, these figures increased also for thoracic and spinal injuries among the survivors (+30.3%; p = 0.0017; +50.6%; p < 0.0252) (Table 4).

**Table 4:** Trend of the figures and percentages of TBI casualties with injury MAIS ≥ 3 in other body regions according to their survivor/deceased status.

RTC Victims with TBI		TBI Survivors		TBI Non Survivors		Trend (% victims with MAIS ≥3 Injury in other body region per TBI victim)	
Number of TBI victims with MAIS ≥ 3 injuries in other body regions (%)		1996–2001	2003–2008	1996–2001	2003–2008	Survivors	Non Survivors
		*5 044*	*3 134*	*514*	*241*		
Thorax	MAIS 3	211	163	50	36	*+30*.3%***	*+19*.1%*
	MAIS 4	120	104	85	63		
	MAIS 5	5	5	72	42		
	MAIS 6	-	-	58	7		
		***336 (6*.*7%)***	***272 (8*.*7%)***	***265 (51*.*6%)***	***148 (61*.*4%)***		
Abdomen	MAIS 3	50	29	33	13	***-21*.*5%***	*+60*.0%**
	MAIS 4	32	10	22	33		
	MAIS 5	2	2	20	9		
	MAIS 6	-	-	1	2		
		***84 (1*.*7%)***	***41 (1*.*3%)***	***76 (14*.*8%)***	***57 (23*.*7%)***		
Spine and Spinal cord injury	MAIS 3	51	46	12	19	*+50*.6%*	*+177*.9%***
	MAIS 4	1	3	0	0		
	MAIS 5	10	9	9	18		
	MAIS 6	-	-	12	6		
		***62 (1*.*2%)***	***58 (1*.*9%)***	***33 (6*.*4%)***	***43 (17*.*8%)***		
Lesions in other body regions	MAIS 3	488	252	160	88	*-17*.5%*	***+7*.*5%***
	MAIS 4	11	4	26	14		
	MAIS 5	0	0	16	1		
	MAIS 6	-	-	3	0		
		***499 (9*.*9%)***	***256 (8*.*2%)***	***205 (39*.*9%)***	***103 (42*.*7%)***		
**Total** ^**$**^		**808 (16.0%)**	**487 (15.5%)**	**392 (74.3%)**	**196 (81.3%)**	**-3.0%**	+9.4%*

* means p < 0.05 for the corresponding test,

** means p < 0.01,

*** means p < 0.001

^$^ one patient can have multiple lesions at different body regions (polytraumatism)

### Unchanged Sex ratio and increasing in age increased

The sex ratio (SR) displayed a male overrepresentation of 2.7, which was not different between survivors and non-survivors in the period following 2002. There was no significant change in the SR among the TBI survivors or deceased between periods (Table 5).

**Table 5:** Trend of Sex Ratio (Male/Female), age (Mean ± SD) and safety device use among road users in the two observation periods

		Survivors		Non Survivors	
		1996–2001	2003–2008	1996–2001	2003–2008
**Sex ratio**					
N		5041	3112	514	241
Car occupants		1.8	1.9	2.6	1.9
Powered Two-Wheelers		6.9	7.2	16.0	8.0
Pedal Cyclists		4.6	5.0	11.0	15.0
Pedestrians		1.4	1.6	1.6	1.4
Others		6.8	2.4	4.0	NA
**Overall Sex Ratio**		**2.4**	**2.7**	**2.9**	**2.7**
**Age (years) mean ± SD**					
Car occupants		33 ± 16	38 ± 19*	38 ± 19^£^	38 ± 20
Powered Two-Wheelers		28 ± 12	27 ± 12	30 ± 11	31 ± 14^£^
Pedal Cylists		27 ± 19	31 ± 21*	46 ± 26^£^	43 ± 23^£^
Pedestrians		36 ± 25	38 ± 26*	53 ± 26^£^	61 ± 24^£^
Others		31 ± 18	34 ± 20	38 ± 21	49 ± 9^£^
**Mean**		**32 ± 18**	33 ± 19*	**41 ± 22**^£^	**44 ± 23**^£^
**Seat belt or Helmet wearing (N)**					
Car occupants ^$^	Yes	1380	845	66^£^	14^£^
	No	602	206	73	12
Powered Two-Wheelers ^$^	Yes	539	521	35^£^	36
	No	110	124	13	10

^£^ means p < 0.05 betwe en deceased and survivors in a given period

* means p < 0.05 between periods

^$^ this information was not available in every road casualty. Consecutively, the sum of figures are below those observed in Table 3.

The SR was the highest for PTW and pedal cyclists and even more so among the deceased for these road user types. The average age of the casualties was 10 years higher for the deceased TBI casualties compared to the survivors, in both periods. Age increased significantly in the period 2003–2008 (p < 0.0001), except for PTW users (Table 5). Pedestrians were significantly older and PTW users significantly younger than other road users (p < 0.001).

**Fig 2:**
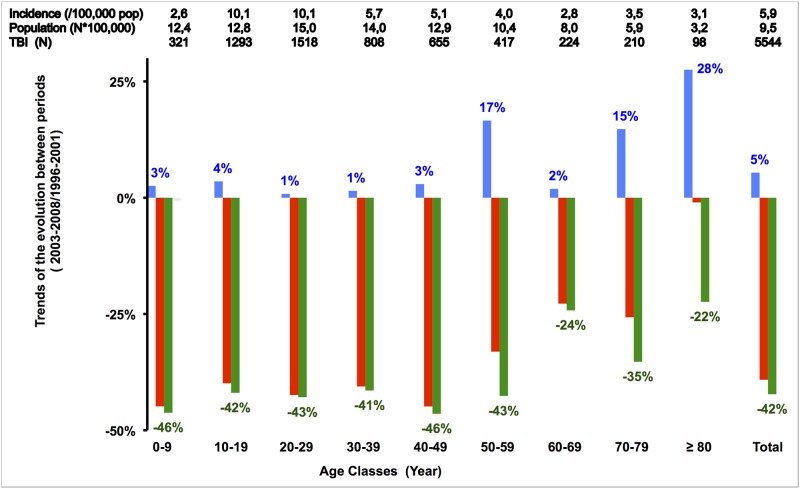
Trend of epidemic of RTC-related TBI incidence by age classes. Trends between the earlier (1996–2001) and later (2003–2008) periods, for 1/ population (blue bars), 2/ number of RTC-related TBI (red bars) and 3/ RTC-related TBI incidence (green bars), divided by 10-year age classes. For the period 1996–2001, the exact numbers are also provided at the top of the graph. This Figure can be read as follows: for the period 1996–2001, in the age class 0–9 years, population was 12.4 (*100,000) inhab., there were 321 TBI victims, and TBI incidence was thus 2.6/100,000 inhab. When comparing with the later period, we observed a population increase of 3%, while the number of RTC-related TBI decreased 45%, and RTC-related TBI incidence decreased 46%.

There was an overall 43% reduction in the mean TBI incidence for all 10-year age classes below 60 in the period 2003–2008. The decrease declined for older classes (Fig 2) when comparisons were made between periods. The reduced TBI incidence observed among the oldest classes was marked by a double effect: a weak reduction in the number of TBIs and an increase in the number of inhabitants in these age class groups in comparison to younger age classes.

### Non-use of seatbelts and helmets was associated with TBI and TBI fatality

The percentage of helmet-wearing was lower among PTW users who sustained TBI (83% and 81% in 1996–2001 and 2003–2008, respectively) in comparison with the national survey figures of helmet-use on PTWs, which displayed a very high rate of helmet-wearing (> 95%)(14). This percentage was even lower among deceased PTW users (73% and 75% in both periods respectively, p < 0.001 in comparison with survivors).

Similarly, the seat-belt wearing rate, which is >90% in nationwide surveys, was lower for motorists who survived a road crash but sustained a TBI (70%, increasing to 80% during the period 2003–2008). These figures dropped to 47% and 53% in the earlier and later period respectively among RTC-induced TBI fatalities (data not shown).

### Survival time and neurosurgical unit (NSU) admission

**Fig 3:**
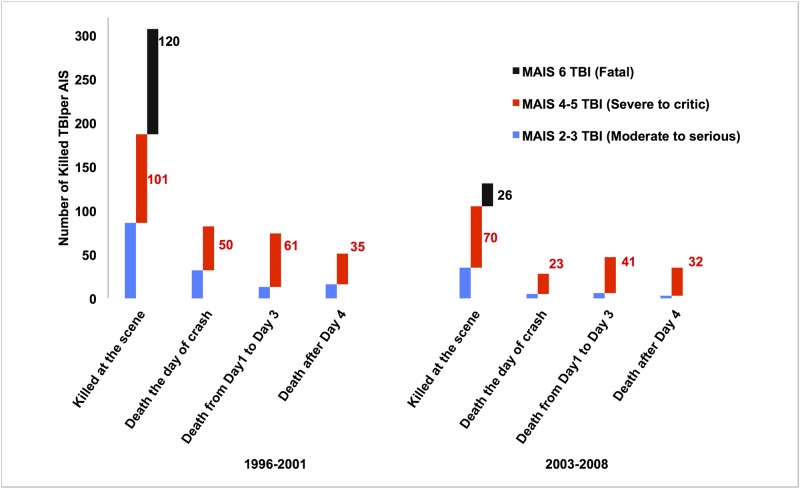
Number of RTC-related TBI victims who deceased in the two periods according to their head-MAIS status and the delay from crash to death

The main share of killed TBI victims in the two periods died within the first 24h after the crash, i.e. at the scene or during the first day after RTC (Fig 3). This type of TBI-related death constituted 76% of all deaths in the 1996–2001 period, dropping to 66% in the 2003–2008 period. In the same time frame, there was a 5% increase of the proportion of death between 1 to 3 days post-TBI and a 5% decrease after Day 4. Interestingly, if we only take into account the victims who are still alive when they arrive at hospital but die thereafter, the share of RTC victims with moderate to serious TBI shrunk the most (-56%), with the group of severe to critical TBI victims dropping only 24% between the period 1996–2001 and the period 2003–2008 (p < 0.001). This was not associated with a significant modification of admission in the neurosurgical unit. In the period 1996–2001, 190 of the 649 AIS 4 and 5 TBI casualties were transferred to a NSU (29.2%) and 140 of the 543 (25.8%) in the period 2003–2008.

### Risk factors for death

In the multivariate analysis, the period 2003–2008 still had a protective effect on the risk of death after an RTC-related TBI (OR = 0.52 [0.41–0.67]; p < 0.0001) after adjustment for TBI severity, ISS, age, sex and road user type. As expected, the risk of death rose when age increased, particularly for victims over 60 and even more for those over 80 (OR = 11.41 [6.72–19.37], p < 0.0001). In addition, the increased severity of TBI (OR = 13.24 for AIS 3 and OR = 76.43 for AIS 5) or Injury Severity Score, without taking into account TBI lesions, was associated with the greatest increase in the risk of death (p < 0.0001 for global effects of both head injury and ISS). Neither sex nor road user type had a significant effect on the change in the risk of death between periods, although they reached significance in the univariate analyses (Table 6).

**Table 6:** Logistic regression analysis of the odds ratio of TBI-related deaths after RTC, adjusted for age, sex, period, AIS severity of the TBI and ISS excluding the head trauma.

Odds Ratio Estimates of death [95% Confidential Interval]			
Factors	Univariate analysis	Multivariate analysis	*p* *For multivariate analysis*
Period			*< 0*.*0001*
1996–2001	Reference	Reference	
2003–2008	0.76 [0.65–89]	0.52 [0.41–0.67]	
Age			*< 0*.*0001*
< 50y	Reference	Reference	
50-59y	1.42 [1.08–1.86]	1.49 0.97–2.27]	
60-69y	1.99 [1.46–2.71]	1.81 [1.10–2.98]	
70-79y	3.43 [2.61–4.49]	3.95 [2.58–6.06]	
>80y	6.01 [4.38–8.26]	11.41 [6.72–19.37]	
Sex			*0*.*274*
Male	Reference	Reference	
Female	1.10 [0.93–1.30]	1.17 [0.88–1.55]	
TBI severity			*< 0*.*0001*
MAIS 2	Reference	Reference	
MAIS 3	31.25 [22.47–43.44]	13.24 [9.14–19.18]	
MAIS 4	44.32 [32.22–60.96]	16.99 [11.86–24.33]	
MAIS 5	154.5 [110.1–216.9]	76.43 [52.14–112.0]	
MAIS 6	NA	NA	
Injury Severity Score (Without Head Injury)			*< 0*.*0001*
< 9	Reference	Reference	
9 to 16	4.67 [3.62–6.01]	2.48 [1.78–3.45]	
17 to 25	16.45 [12.68–21.35]	5.99 [4.26–8.43]	
>25	82.14 [64.81–104.11]	33.17 [24.14–45.58]	
Road Users			*0*.*0814*
Car occupants	Reference	Reference	
Powered Two-Wheelers	1.04 [0.85–1.28]	0.91 [0.66–1.25]	
Cyclists	0.39 [0.28–0.55]	0.61 [0.38–0.99]	
Pedestrians	1.78 [1.48–2.14]	1.21 [0.88–165]	
Others	0.82 [0.56–1.22]	1.28 [0.70–2.34]	

## Discussion

### Statement of principal findings

Despite identification of road trauma as one of the leading causes of death or disability among non-transmitted diseases in the Global Burden of Disease project [2, 3], no details are provided on the type of lesions endured by traumatized patients. Thus, despite the fact that TBI is the most damaging form of trauma, it is virtually impossible to identify in such data, in particular when relating to traumatic brain injury. TBI is now considered to leave people with minor to major sequels in more than 50% of cases, this number increasing with the severity of the brain lesions [9, 15–20]. In Western countries, road crash induced TBIs account for 11% to 60% of all TBIs, reaching 50% in France [1–3, 6, 8, 9, 15–26]. To allocate care and funds, prevention and effective treatment for brain-injured victims requires knowledge of the TBI epidemic. For this reason, the Registry of RTC trauma patients was created more than 20 years ago, in the area of Lyon, France. It aimed to detail the physical trauma of people sustaining RTC and depicting the trends of RTCs and their health burden over the course of time. In 2002, a large range of legislative measures pertaining to road safety was introduced in France (Table 7). Notably, between 2001 and 2008, there was a 9-fold increase in prosecutions related to speed controls. [15].

**Table 7:** law enforcement timetable since 2002 in France

Year	Measures
2002	The French President announces that road safety is to be one of the 3 main priorities of his term of office
2003	Automatic speed enforcement
	First automatic radar speed traps
	Stiffer penalties for unintentional manslaughter and wounding
	Stiffer penalties (drink-driving, not wearing a safety belt or helmet, mobile telephone use)
	Stiffer penalties for re-offending
	Systematic drugs screening of crash-involved drivers
	Safety belt wearing made compulsory for drivers of HGVs and coaches
	Introduction of a probationary driving licence for young drivers
2004	Reduction of the legal limit for alcohol for public transport vehicle drivers to 0.2g/l
	Introduction of specific penalties for exceeding the speed limit by over 50 km/h
2005	Speed limiting devices made compulsory for HGVs weighing over 3.5 tonnes and public transport vehicles weighing over 10tonnes
	Legal obligation for drivers to make sure that any minors they are carrying have fastened their safety belt

The present study, which compares the 6-year periods before and after the strengthened legislation and enforcement, provides us with 4 main results. Firstly, there was a dramatic 56% drop in RTC-related TBI mortality rate (the ratio killed victims of TBI/population in a given period) between the two periods, whereas RTC incidence was reduced only 25%. Secondly, there was a 42% reduction of the incidence of TBI casualties related to RTC in the 2003–2008 period, compared to the earlier period (mainly among MAIS-2, -3 and -6 TBI). Consequently, the TBI lethality rate (the ratio of deceased TBI among TBI victims) decreased 23.5% in the period 2003–2008. Thirdly, these trends affected more car occupants and less PTW users, which could at least partly explain changes in associated body injuries. The stark reduction in the involvement of car occupants in the period 2003–2008 could be explained by the interference of several factors: legislative changes, resulting in behavioural changes among car users, improvements in primary safety of the car fleet (i.e. seatbelt pre-tensioners and airbag systems for all car occupants have been mandatory for all new care sales since 1998), improvements in road infrastructure safety (which were calibrated for car users, who form the bulk of road users), improvements in medical care at the emergency pre-hospital, hospital settings or thereafter. Fourthly, after adjusting for sex, age, severity of injuries to the head and to the rest of the body, and road user type, the TBI odds ratio of death remained reduced in the period 2003–2008. This points to the effects of legislative changes on road user behaviours (Table 7), as well as improvements in the health care system for RTC-related TBI victims.

### Strengths and weaknesses in relation to other studies

According to the department of transport in the UK, the lethality rate among RTC victims increased 10% in Britain. In the Rhône Registry, the lethality rate among RTC victims dropped 19%, from 1.2% to 1.0% death/RTC for the same periods (Table 8).

**Table 8:** Comparison of epidemics of RTC in United Kingdom, France and the Rhone County in the two periods studied

	1996–2001			2003–2008			
	Road traffic casualties	Death	Lethality rate	Road traffic casualties	Death	Lethality rate	Trends in Lethality rate
UK	1,906,595	20,900	1.1%	1,332,600	16,048	1.2%	10%
Rhône	65,519	793	1.2%	50,239	508	1.0%	-19%
France	991,864	47,898	4.8%	631,495	30,246	4.8%	0%

Thus, since the introduction of the recent legislative measures in France, we have observed a more marked reduction in the incidence of killed victims of RTC in the Rhône area or France compared to the UK.

RTC data are mainly recorded by the police forces and centralized by the Departments of Transport in France [15], the USA [10] and the UK [11]. In contrast to police data, the Registry is the result of a collaborative medical work of more than 245 health care services. Interestingly, according to French national police records, the lethality rate among RTC victims between the periods 1996–2001 and 2003–2008 remained stable around 4.8% [15]. According to French and UK national figures, there are two times less recorded RTC victims in France than in the UK in both periods. This could be partly due to the documented under-reporting of RTC by the French police services [27,28]. For instance, this under-reporting has reached 60% in the Rhone area, particularly when RTC involves victims with low severity injuries, pedal cyclists and PTW users, and when RTC occurs without a third party [27,28]. Moreover, the data from the Department for Transport Statistics, obtained in the period 2003–2008 in the UK, was combined and cross-checked with the National Health Service records; this could have resulted in the apparent increase in injured victims recorded in this last period.

In addition, not only can any study based on police crash data be misleading, but this data cannot identify the epidemic of TBI, and must be combined with medical/hospital registries. The comparison between New Zealand and UK data highlights the importance of a comprehensive recording of TBI to explain the incidence, trends and prognosis in a given population [24].

In the present study, the incidence of RTC fatality occurring in the presence of a TBI during the 2003–2008 period was 33.9/100,000 inhabitants, which corresponds to a 25% decrease when compared to the previous period. This incidence was significantly lower than that reported in other Western and European countries [1,10,11,29,30,31]. Our results are likely to be partly due to the fact that the Rhône County is highly urbanized—a state, which generally leads to, fewer accidents compared with more rural French counties or other European countries [30,31]—and to the limited field of this study to RTC-related TBI. Interestingly, in the Registry, the share of deceased victims with a TBI has decreased from 65% (514/793) to 49% (241/504) (p < 0.001). This might have been associated not only with the reduction of RTC-related TBI incidence, but also with changes in the early medical management of RTC victims, by performing full body CT-scans in all RTC victims involved in a high velocity crash, as is now recommended [32]. Indeed, TBI lesions assessment is now more accurate, because they are identified as anatomical lesions observed by the CT-scan, and no longer only through abnormal level of consciousness, which may be caused by other pathological conditions.

In our study, during the 1996–2001 period, the mortality rate in TBI patients (9.2%) was similar to that registered in other studies covering the 2000–2005 [5, 8, 9, 21, 29] or 1997–2007 [1, 9] periods, while some authors included AIS 3–5 casualties only [9,21,22,24,33,34]. When we restricted our analysis to these severities of casualties only, the Rhone Registry exhibited a mortality rate of 31.5% for the period 1996–2001 that decreased to 24.3% during the period 2003–2008. Other methodological limitations in previous studies can explain the differences with our results, such as the inclusion of isolated TBI only [24], age restriction to >14 years [32], lack of information on pedestrians [33,34], and inclusion of patients reaching the facility cares but not those dying at the scene [8,21,22,25,34,35]. When focusing on trends, the survey published on the TBI epidemics in the USA announced a 22% decrease in mortality rates between 1997 and 2007 [1]; however, this shrinks to a 10.5% reduction when comparing the two 5-year periods, before and after 2002 –a 5 times smaller reduction than that observed in the present study.

Using nationwide hospital charts and the registry of death in Finland, Koskinen et Alaranta found a 7% increase in TBI incidence in Finland but pointed out that RTC-related TBI (which accounts for 20% of TBI in the 1991–1995 period), decreased 20% in the 2001–2005 period in comparison to 10 years earlier. Although TBI mortality rates from all causes were reduced 32.2% between 1992 and 2002, they did not calculate the nationwide mortality rate of the main TBI aetiology [29]. Paralleling these results, the TARN group observed an annual decrease of 3% for TBI mortality in England and Wales between 1989 and 1994, a decrease which was not sustained during the remaining years of observation (1995–1999). However, the TARN studies also did not mention the modifications of the TBI aetiology [8, 21, 22]. By this time, the TARN authors concluded that this improvement was due either to changes in standards of emergency care [8], or to an increased rate of transfer to neurosurgical units for TBI patients [21–24]. However, as far as we know, there have been no changes in international standards of TBI management, which could account for the 56% decrease in the TBI mortality rate or the 22.8% reduction in the lethality rate observed in the present study. By the same token, we did not observe any significant change in the share of TBI patients undergoing treatment in the NSU, nor in the number of killed TBI patients who died after the first initial days following the crash (see Fig 3). Considering that primary safety of the car fleet and roads, as well as standards of care for TBI have sprouted similarly in the developed world, nowhere else than in France have the drops in TBI mortality and RTC related TBI incidence displayed the same magnitude of reduction.

Thus, we can definitely hypothesize that legislative changes introduced in France since 2002 have significantly participated in the change of the human factors on roads, and have led to the reduction of the severity of road crashes, resulting in a change in TBI epidemics. These effects cannot be explained only by the improvement of either pre-hospital and hospital care or prevention systems in or outside the vehicles. From a logical standpoint, there is no reason to think that improved medical care would affect the incidence of TBI, as car crashes happen before medical care is provided. On the other hand, the lethality rate is directly influenced by the quality of care, which may differ from one country to the next. These effects also could be explained by the reduction in driving speed and other impacts related to human factors [4]. One of author in our group has previously published a study, showing that speeding decreased significantly on secondary roads in France over the period 2001–2010. As most fatal car accidents occur on this type of road, the effect of reducing speed was directly associated with the decrease in mortality rate [36]

### Road Users

According to UK, USA and French authorities [1, 10, 11, 15], the trends in road crash incidence depicted a 21% reduction in France, close to that observed in the UK (-18%), and higher than that in the USA (-1%). As in the UK, we observed a stable incidence in PTW-RTC (-1%) in the Registry, but far from those observed in the USA (+53%). These differences in the trends were similar when compared to the killed PTW users in the Rhone County (-26%) and in the UK (-23%), which were much lower than in the USA (+83%) for the same periods [10–11].

Interestingly, although the overall figures, the incidences and TBI mortality of motorcycle riders and pedal cycle RTC decreased in the recent period, AIS-4 and -5 TBI casualties in these two road user categories slightly increased in the recent period in the Rhone County. Associated with an increase in body lesion severity, these results suggest also that a change in road user behaviour has progressively occurred over the two periods, the car occupants switching from car driving to PTW or cycle riding. This is confirmed by the motorbike and motorcycles sales, which have doubled in France in the period 1996–2007 (a figure similar to that observed in the UK). Among other reasons, this may have been due to the fact that people already licensed for car-driving could drive a motorcycle under 125cc without another specific license category, and also that the speed cameras initially installed in France flashed frontally only, and therefore were unable to capture two-wheel users.

While only 3.7% of the 13,000 respondents to a Rhone county survey on transport use reported using a motorcycle [24]—a result similar to those observed in other countries [37] - 25% of RTC deaths involved PTW [15]. This illustrates that PTW drivers are thus disproportionately represented among RTC casualties.

The increased number of AIS-4 and AIS-5 TBI casualties, which were observed among pedal cyclists during the period 2003–2008, may be partly related to an increase in bicycle traffic. The use of bicycles as a means of transportation has massively increased during this period, following the introduction of a self-service bicycle hire system in June 2005 [38,39]. The reduction in RTC pedal cyclist numbers, whether less than this for car occupants, was of the same magnitude as that observed in the UK (-26%), but greater than in the USA (-5%), for the same periods. Therefore, if one consider that these reductions in RTC may be connected to legislative measures, it is clear that these measures had a much weaker impact on PTW and pedal cyclists than they did on car occupants.

### Non-cranial associated injuries

The number of TBI victims with lesions in other body regions than the head increased in the 2003–2008 period and especially among the TBI patients who did not survive. Our data for killed victims after TBI are in accordance with others, who showed that non-cranial injury occurrence increased the risk of early mortality [40]. This increase in severe extra-cranial injuries might be explained either by a change in RTC victim recruitment (increased share of pedal cyclists and/or PTW users), or by modifications in procedures allowing a more sensitive diagnosis of TBI (i.e. recommendation of total body CT-scan for all patients injured following a high-velocity Trauma)[32]. However, the multivariate analysis we performed found the effect of the period to remain significant, taking into account the severity of injuries at the head and in other body regions.

In comparison to other studies [1,21–26], our accurate evaluation of TBI mortality rates after RTC, although focused on a limited geographical zone, is strengthened by the exhaustive inclusion of all RTC, and consequently all TBI casualties, whatever their severity. This comprehensive coverage and follow-up has been used to correct and complete the national data obtained from police services [27,28]. Moreover, the inclusion of victims dying at the scene allows for a more accurate analysis of the evolution of the odds ratio of death between periods. While most of the studies only include AIS 3 to AIS 5 TBI casualties, we extended inclusions in the Registry to those who deceased before reaching care facilities and those sustaining AIS 2 injuries. The inclusion of AIS 2 TBI, defined at least by a loss of consciousness of more than 5 minutes, is justified by the fact that it is suspected to contribute to today's epidemic of Post-Traumatic Stress Disorder and post-concussion syndrome whose cost amounts to millions of dollars a year in the US [35].

Considering the TBI epidemic, our study is limited to those resulting from RTC, which represented until recently about 50% of severe TBI casualties in France [32].

In addition, it could be argued that a long duration Registry could lead to drop outs from collaborator participation, and then to a decrease in the amount of cases recorded, particularly among low-severity brain trauma casualties (AIS 2 TBI). However, when the results from different institutions contributing to the Registry or national survey are compared (data not shown), the consistency in the observed trends suggests that they do not depend on the level of medical collaboration. Although this study did not consider one-year outcomes following head trauma, it did record mortality, and the quality of the scoring system we used (a combination of GCS and AIS) has been shown to correlate well with the GOS scale [41,42]. When recruitment began (in 1995), the Registry did not include information on the ethnicity or the socioeconomic status, even if such information is now considered important for the understanding of TBI and the road traffic collision epidemic [23,43–46]. Finally, the present study did not have the means to identify, among the following legislative measures, what most contributes to these changes in TBI morbidity and mortality related to RTC. These changes could result from the enforcement and combination of measures relating to 1) car manufacturing to, 2) road transport infrastructure and 3) road user behaviour.

### Meaning of the study: implication for policy makers

In this French study, conducted in the Rhone Department, RTC-related TBI incidence was reduced almost two times more than RTC casualty incidence in the more recent period. This decrease concerned all TBI AIS severities and was less marked for severe and critical TBI than for either fatal or minor and serious TBI. The fact that the incidence of severe and critical TBI dropped less than the incidence of all TBI was associated with increases in the share of two-wheelers, bicyclists and pedestrians represented among TBI casualties. However, we observed an increase in age and in associated body injuries per casualty, particularly among the deceased. All of these results suggest that TBI casualties are more affected by law enforcement than other body region casualties, and that TBI casualties are now more likely than before to involve vulnerable road users (PTW users, pedal cyclists and pedestrians). If we put back-to-back international changes observed in TBI epidemics, deriving from RTC in developed countries, and the RTC and TBI figures observed for the Rhone area, we can conclude that the legislative measures gradually introduced in France since 2002 were strikingly effective for car users, but less so for PTW users, pedal cyclists and pedestrians. Further legislative improvements may thus be needed to gain impacts on these categories of road users. Finally, we encourage politicians of countries with high levels of RTC, first, to correctly identify the most at-risk groups by means of a registry, and then to target these groups by introducing appropriate legislative measures.

Further studies may help in defining new legislative tools to keep up this positive trend of RTC-related TBI reductions in the near future—namely, research on the effects of neurosurgical admission, and of the detailed analysis of CT-scans for each brain lesion. Follow-up studies on TBI-survivors in the years after their hospitalization, as well as analyses of the RTC-related TBI epidemic in the current 6-year period are in progress, and can be expected to greatly improve our understanding and approaches towards RTC-related TBI.

## Supporting Information

S1 Fig Data sheet fllled for each patient at the time of his interaction with the health care system. The same patient may have different sheets depending on number of health care service where he is admitted (i.e. when a patient is transfered from the emergency room to a surgical ward requires that 2 different sheets are completed: one from the emergency room and one from the surgical ward).
(TIFF)

## References

[1] Coronado VG, Xu L, Basavaraju SV, McGuire LC, Wald MM, Faul MD, et al Centers for Disease Control and Prevention (CDC). Surveillance for traumatic brain injury-related deaths—United States, 1997–2007. MMWR Surveill Summ. 2011 5 6;60(5):1–32. 21544045

[2] Lozano R, Naghavi M, Foreman K, Lim S, Shibuya K, Aboyans V, et al Global and regional mortality from 235 causes of death for 20 age groups in 1990 and 2010: a systematic analysis for the Global Burden of Disease Study 2010. Lancet. 2012 12 15;380(9859):2095–128. Lancet 2012 380:2095–2128 10.1016/S0140-6736(12)61728-0 23245604 PMC10790329

[3] Murray CJ, Atkinson C, Bhalla K, Birbeck G, Burstein R, Chou D, et al; U.S. Burden of Disease Collaborators. The state of US health, 1990–2010: burden of diseases, injuries, and risk factors. JAMA. 2013 8 14;310(6):591–608. 10.1001/jama.2013.13805 23842577 PMC5436627

[4] Laumon B, Gadegbeku B, Martin JL, Biecheler MB; SAM Group. Cannabis intoxication and fatal road crashes in France: population based case-control study. BMJ. 2005 12 10;331(7529):1371 Epub 2005 Dec 1. 10.1136/bmj.38648.617986.1F 16321993 PMC1309644

[5] Hetland A, Carr DB. Medications and impaired driving. Ann Pharmacother. 2014 4;48(4):494–506. Epub 2014 Jan 28. 10.1177/1060028014520882 24473486 PMC3965581

[6] Pilkington P, Kinra S. Effectiveness of speed cameras in preventing road traffic collisions and related casualties: systematic review. BMJ. 2005 2 12;330(7487):331–4. Epub 2005 Jan 14. 10.1136/bmj.38324.646574.AE 15653699 PMC548724

[7] Blomberg RD, Peck RC, Moskowitz H, Burns M, Fiorentino D. The Long Beach/Fort Lauderdale relative risk study. J Safety Res 2009;40 (4):285–92 10.1016/j.jsr.2009.07.002 19778652

[8] Lecky F, Woodford M, Yates DW. Trends in trauma care in England and Wales 1989–97. UK Trauma Audit and Research Network. Lancet. 2000;355(9217):1771–5. 10832827 10.1016/s0140-6736(00)02264-9

[9] Masson F, Thicoipe M, Aye P, Mokni T, Senjean P, Schmitt V, et al; Aquitaine Group for Severe Brain Injuries Study. Epidemiology of severe brain injuries: a prospective population-based study. J Trauma. 2001;51:481–9. 11535895 10.1097/00005373-200109000-00010

[10] http://www-fars.nhtsa.dot.gov/Main/index.aspx available October 2015

[11] https://www.gov.uk/government/statistics/reported-road-casualties-great-britain-annual-report-2013 or https://www.gov.uk/government/collections/road-accidents-and-safety-statistics available October 2015

[12] Laumon B, Martin JL, Collet P, Chiron M, Verney MP, Ndiaye A, et al, 1997. A French road accident trauma registry: first results. In: 41st Annual Proceedings of the Association for the Advancement of Automotive Medicine, Orlando, Florida, pp. 127–137.

[13] Lieutaud T, Ndiaye A, Laumon B, Chiron M. Spinal cord injuries sustained in road crashes are not on the decrease in france: a study based on epidemiological trends. J Neurotrauma. 2012 10;29(3):479–87. 10.1089/neu.2011.1880 21895531

[14] Association for the Advancement of Automotive Medicine. (1990). The Abbreviated Injury Scale, 1990 Revision. Des Plaines, IL: Association for the Advancement of Automotive Medicine.

[15] http://www.securite-routiere.gouv.fr/media/fichiers/onisr/bilan-du-comportement-des-usagers-de-la-route-annee-2009?xtmc=bilan+annee+2009&xtcr=5.

[16] Mazaux JM, Masson F, Levin HS, Alaoui P, Maurette P, Barat M. Long-term neuropsychological outcome and loss of social autonomy after traumatic brain injury. Arch Phys Med Rehabil. 1997 12;78(12):1316–20. 9421984 10.1016/s0003-9993(97)90303-8

[17] Nash S, Luauté J, Bar JY, Sancho PO, Hours M, Chossegros L, et al Cognitive and behavioural post-traumatic impairments: what is the specificity of a brain injury? A study within the ESPARR cohort. Ann Phys Rehabil Med. 2014 12;57(9–10):600–17. Epub 2014 Sep 16. 10.1016/j.rehab.2014.08.010 25267451

[18] Dikmen SS, Machamer JE, Powell JM, Temkin NR. Outcome 3 to 5 Years After Moderate to Severe Traumatic Brain Injury. Arch Phys Med Rehabil 2003;84:1449–57. 14586911 10.1016/s0003-9993(03)00287-9

[19] Machamer J, Temkin N, Dikmen S. Health-Related Quality of Life in Traumatic Brain Injury: Is a Proxy Report Necessary? J Neurotrauma 2013;30:1845–51 10.1089/neu.2013.2920 23731370 PMC3814818

[20] Temkin NR, Anderson GD, Winn HR, Ellenbogen RG, Britz GW, Schuster J, et al Magnesium sulfate for neuroprotection after traumatic brain injury: a randomised controlled trial. Lancet Neurol 2007; 6: 29–38 10.1016/S1474-4422(06)70630-5 17166799

[21] Patel HC, Bouamra O, Woodford M, King AT, Yates DW, Lecky FE; Trauma Audit and Research Network. Trends in head injury outcome from 1989 to 2003 and the effect of neurosurgical care: an observational study. Lancet. 2005;366(9496):1538–44. 10.1016/S0140-6736(05)67626-X 16257340

[22] Fuller G, Bouamra O, Woodford M, Jenks T, Patel H, Coats TJ, et al Temporal trends in head injury outcomes from 2003 to 2009 in England and Wales. Br J Neurosurg. 2011;25(3):414–21. 10.3109/02688697.2011.570882 21513451

[23] Feigin VL, Theadom A, Barker-Collo S, Starkey NJ, McPherson K, Kahan M, et al BIONIC Study Group. Incidence of traumatic brain injury in New Zealand: a population-based study. Lancet Neurol. 2013;12:53–64. 10.1016/S1474-4422(12)70262-4 23177532

[24] Gabbe BJ, Lyons RA, Lecky FE, Bouamra O, Woodford M, Coats TJ, et al Comparison of mortality following hospitalisation for isolated head injury in England and Wales, and Victoria, Australia. PLoS One. 2011;6(5):e20545 Epub 2011 May 31. 10.1371/journal.pone.0020545 21655237 PMC3105093

[25] Tagliaferri F, Compagnone C, Korsic M, Servadei F, Kraus J. A systematic review of brain injury epidemiology in Europe. Acta Neurochir (Wien). 2006;148(3):255–68 16311842 10.1007/s00701-005-0651-y

[26] Edwards P, Arango M, Balica L, Cottingham R, El-Sayed H, Farrell B, et al Final results of MRC CRASH, a randomised placebo-controlled trial of intravenous corticosteroid in adults with head injury-outcomes at 6 months. Lancet. 2005;365(9475):1957–9. 10.1016/S0140-6736(05)66552-X 15936423

[27] Amoros E, Martin JL, Laumon B. Under-reporting of road crash casualties in France. Accid Anal Prev. 2006 7;38(4):627–35. Epub 2006 Mar 20. 10.1016/j.aap.2005.11.006 16545764

[28] Amoros E, Martin JL, Lafont S, Laumon B. Actual incidences of road casualties, and their injury severity, modelled from police and hospital data, France. Eur J Public Health. 2008 8;18(4):360–5. Epub 2008 Mar 31. 10.1093/eurpub/ckn018 18381295

[29] Koskinen S, Alaranta H. Traumatic brain injury in Finland 1991–2005: a nationwide register study of hospitalized and fatal TBI. Brain Inj. 2008;22:205–14 10.1080/02699050801938975 18297592

[30] Clark DE. Effect of population density on mortality after motor vehicle collisions. Accid Anal Prev. 2003;35(6):965–71. 12971931 10.1016/s0001-4575(02)00104-5

[31] Eksler V, Lassarre S, Thomas I. Regional analysis of road mortality in Europe. Public Health. 2008;122:826–37. 10.1016/j.puhe.2007.10.003 18620716

[32] Yeguiayan JM, Yap A, Freysz M, Garrigue D, Jacquot C, Martin C, et al Impact of whole-body computed tomography on mortality and surgical management of severe blunt trauma. Crit Care. 2012 6 11;16(3):R101 10.1186/cc11375 22687140 PMC3580653

[33] Berry C, Ley EJ, Tillou A, Cryer G, Margulies DR, Salim A. The effect of gender on patients with moderate to severe head injuries. J Trauma. 2009;67(5):950–3. 10.1097/TA.0b013e3181ba3354 19901653

[34] Brown JB, Stassen NA, Cheng JD, Sangosanya AT, Bankey PE, Gestring ML. Trauma center designation correlates with functional independence after severe but not moderate traumatic brain injury. J Trauma. 2010 8;69(2):263–9. 10.1097/TA.0b013e3181e5d72e 20699734

[35] Cassidy JD, Carroll LJ, Peloso PM, Borg J, von Holst H, Holm L, et al WHO Collaborating Centre Task Force on Mild Traumatic Brain Injury. Incidence, risk factors and prevention of mild traumatic brain injury: results of the WHO Collaborating Centre Task Force on Mild Traumatic Brain Injury. J Rehabil Med. 2004;(43 Suppl):28–60. 15083870 10.1080/16501960410023732

[36] Viallon V, Laumon B. Fractions of fatal crashes attributable to speeding: Evolution for the period 2001–2010 in France. Accid Anal Prev 2013; 52(3):250–6 23414912 10.1016/j.aap.2012.12.024

[37] Heyes GJ, Craig J, Hinds JD, Kealey DW. The burdein of motorcycle trauma and seasonal change at a regional trauma center. Ulster Med J. 2014;83:55–6 24757276 PMC3992101

[38] Jensen P, Rouquier JB, Ovtracht N, Robardet C. Characterizing the speed and paths of shared bicycle use in Lyon. Transportation Research Part D. 2010;15(8):522–4.

[39] http://www.rhonealpes.fr/467-le-velo-v.htm Available October 2015

[40] van Leeuwen N, Lingsma HF, Perel P, Lecky F, Roozenbeek B, Lu J, et al International Mission on Prognosis and Clinical Trial Design in TBI Study Group; Corticosteroid Randomization After Significant Head Injury Trial Collaborators; Trauma Audit and Research Network. Prognostic value of major extracranial injury in traumatic brain injury: an individual patient data meta-analysis in 39,274 patients. Neurosurgery. 2012;70:811–8. 10.1227/NEU.0b013e318235d640 21904253

[41] Foreman BP, Caesar RR, Parks J, Madden C, Gentilello LM, Shafi S, et al Usefulness of the abbreviated injury score and the injury severity score in comparison to the Glasgow Coma Scale in predicting outcome after traumatic brain injury. J Trauma. 2007 4;62(4):946–50. 10.1097/01.ta.0000229796.14717.3a 17426553

[42] Timmons SD, Bee T, Webb S, Diaz-Arrastia RR, Hesdorffer D. Using the abbreviated injury severity and Glasgow Coma Scale scores to predict 2-week mortality after traumatic brain injury. J Trauma. 2011 11;71(5):1172–8. 10.1097/TA.0b013e31822b0f4b 22071922

[43] Butcher I, McHugh GS, Lu J, Steyerberg EW, Hernández AV, Mushkudiani N, et al Prognostic value of cause of injury in traumatic brain injury: results from the IMPACT study. J Neurotrauma. 2007 2;24(2):281–6. 10.1089/neu.2006.0030 17375992

[44] Braver ER. Race, Hispanic origin, and socioeconomic status in relation to motor vehicle occupant death rates and risk factors among adults. Accid Anal Prev. 2003;35(3):295–309. 12643947 10.1016/s0001-4575(01)00106-3

[45] Grossetête Matthieu. Accidents de la route et inégalités sociales. Les morts, les médias et l’état. Editions du Croquant. 2011. Bellecombe en Bauges.

[46] Licaj I, Haddak M, Pochet P, Chiron M. Contextual deprivation, daily travel and road traffic injuries among the young in the Rhône Département (France). Accid Anal Prev. 2011 9;43(5):1617–23. 10.1016/j.aap.2011.02.003 21658487

