# Supplementary figures and images for: The Decrease in Traumatic Brain Injury Epidemics Deriving from Road Traffic Collision Following Strengthened Legislative Measures in France
Source: PLoS One. 2016 Nov 28;11(11):e0167082. doi: 10.1371/journal.pone.0167082 (PMC5125664; doi:10.1371/journal.pone.0167082)

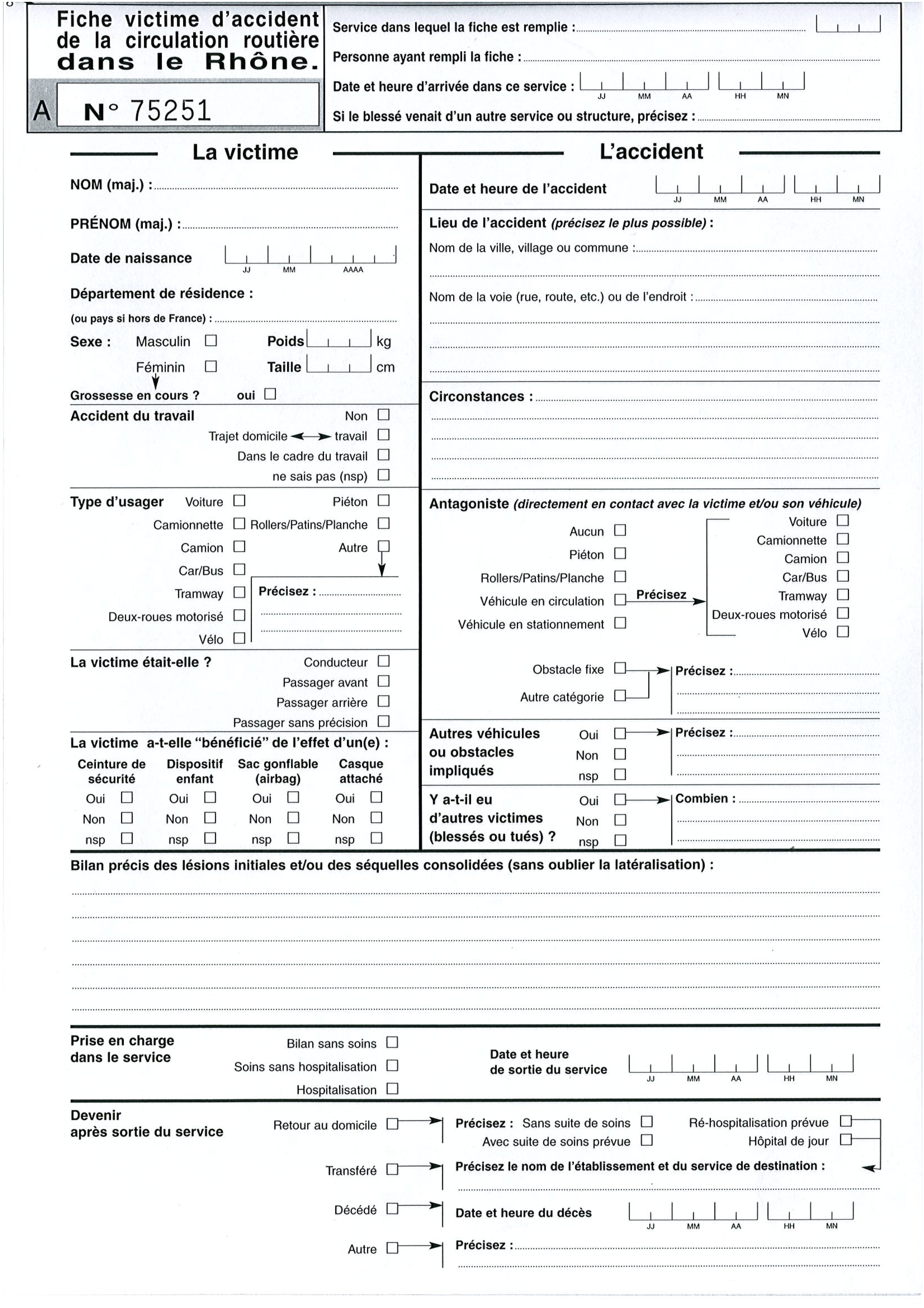

Supplement: S1 Fig — The same patient may have different sheets depending on number of health care service where he is admitted (i.e. when a patient is transfered from the emergency room to a surgical ward requires that 2 different sheets are completed: one from the emergency room and one from the surgical ward). (TIFF) [file pone.0167082.s001.tiff]
